# *trans*-Ethyl­enedi-*p*-phenyl­ene diacetate

**DOI:** 10.1107/S1600536809032620

**Published:** 2009-08-26

**Authors:** Stefanie Ritter, Jörg-M. Neudörfl, Janna Velder, Hans-Günther Schmalz

**Affiliations:** a Department für Chemie der Universität zu Köln, Greinstrasse 4, 50939 Köln, Germany

## Abstract

The centrosymmetric title compound, C_18_H_26_O_4_, was prepared in high yield from 4-acetoxy­styrene *via* Ru-catalysed homo-olefin metathesis. Exclusive formation of the *E*-configurated isomer was observed. In the crystal, a strong C—H⋯π inter­molecular inter­action links the mol­ecules together.

## Related literature

For the preparation of differently substituted stilbenes using a Ru-catalysed metathesis strategy, see: Velder *et al.* (2006 ▶). For alternative methodologies for the synthesis of ­oxy-functionalized stilbenes using Wittig-type olefinations or Heck-couplings, see: Kim *et al.* (2002 ▶); Lion *et al.* (2005 ▶); Botella *et al.* (2004 ▶); Reetz *et al.* (1998 ▶). For the bioactivity of various stilbenes with a focus on their anti­cancer activity, see: Aggarwal *et al.* (2004 ▶); Wolter *et al.* (2002 ▶); Fremont (2000 ▶); Jang *et al.* (1997 ▶); Wieder *et al.* (2001 ▶). For related structures see: Malone *et al.* (1997 ▶). For a previous synthesis of the title compound see: Johnson *et al.* (1952 ▶).
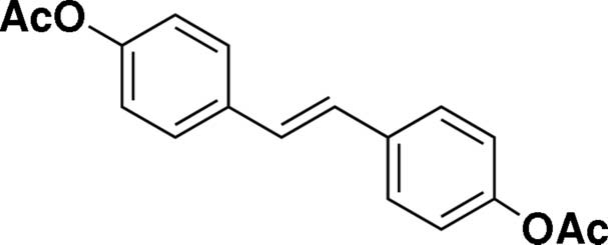


## Experimental

### 

#### Crystal data

C_18_H_16_O_4_
*M* *_r_* = 296.31
Monoclinic, 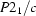

*a* = 9.7430 (4) Å
*b* = 7.2839 (4) Å
*c* = 11.2723 (6) Å
β = 113.649 (3)°
*V* = 732.78 (7) Å^3^
*Z* = 2
Mo *K*α radiation
μ = 0.10 mm^−1^
*T* = 100 K
0.52 × 0.36 × 0.34 mm

#### Data collection

Nonius KappaCCD diffractometer
Absorption correction: none
3533 measured reflections
1595 independent reflections
1119 reflections with *I* > 2σ(*I*)
*R*_int_ = 0.038

#### Refinement

*R*[*F* ^2^ > 2σ(*F* ^2^)] = 0.042
*wR*(*F* ^2^) = 0.106
*S* = 1.03
1595 reflections
101 parameters
H-atom parameters constrained
Δρ_max_ = 0.20 e Å^−3^
Δρ_min_ = −0.21 e Å^−3^

### 

Data collection: *COLLECT* (Hooft, 1998 ▶); cell refinement: *DENZO* (Otwinowski & Minor, 1997 ▶); data reduction: *DENZO*; program(s) used to solve structure: *SHELXS97* (Sheldrick, 2008 ▶); program(s) used to refine structure: *SHELXL97* (Sheldrick, 2008 ▶); molecular graphics: *SCHAKAL99* (Keller, 1999 ▶); software used to prepare material for publication: *PLATON* (Spek, 2009 ▶) and *enCIFer* (Allen *et al.*, 2004 ▶).

## Supplementary Material

Crystal structure: contains datablocks global, I. DOI: 10.1107/S1600536809032620/hg2554sup1.cif

Structure factors: contains datablocks I. DOI: 10.1107/S1600536809032620/hg2554Isup2.hkl

Additional supplementary materials:  crystallographic information; 3D view; checkCIF report

## Figures and Tables

**Table 1:** Geometry the C—H⋯π interaction (Å, °)

*D*—H⋯*A*	*D*—H	H⋯*A*	*D*⋯*A*	*D*—H⋯*A*
C7—H7⋯*Cg*1^i^	0.95	2.81	3.539 (2)	135

Symmetry code: (i) 
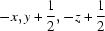
.  *Cg*1 is the centroid of the C2–C7 ring.

**Fig. 1.**
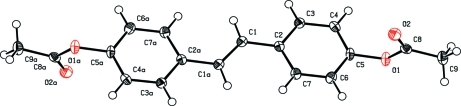
A top view of 1. Displacement ellipsoids are drawn at the 50% probability level.

**Fig. 2.**
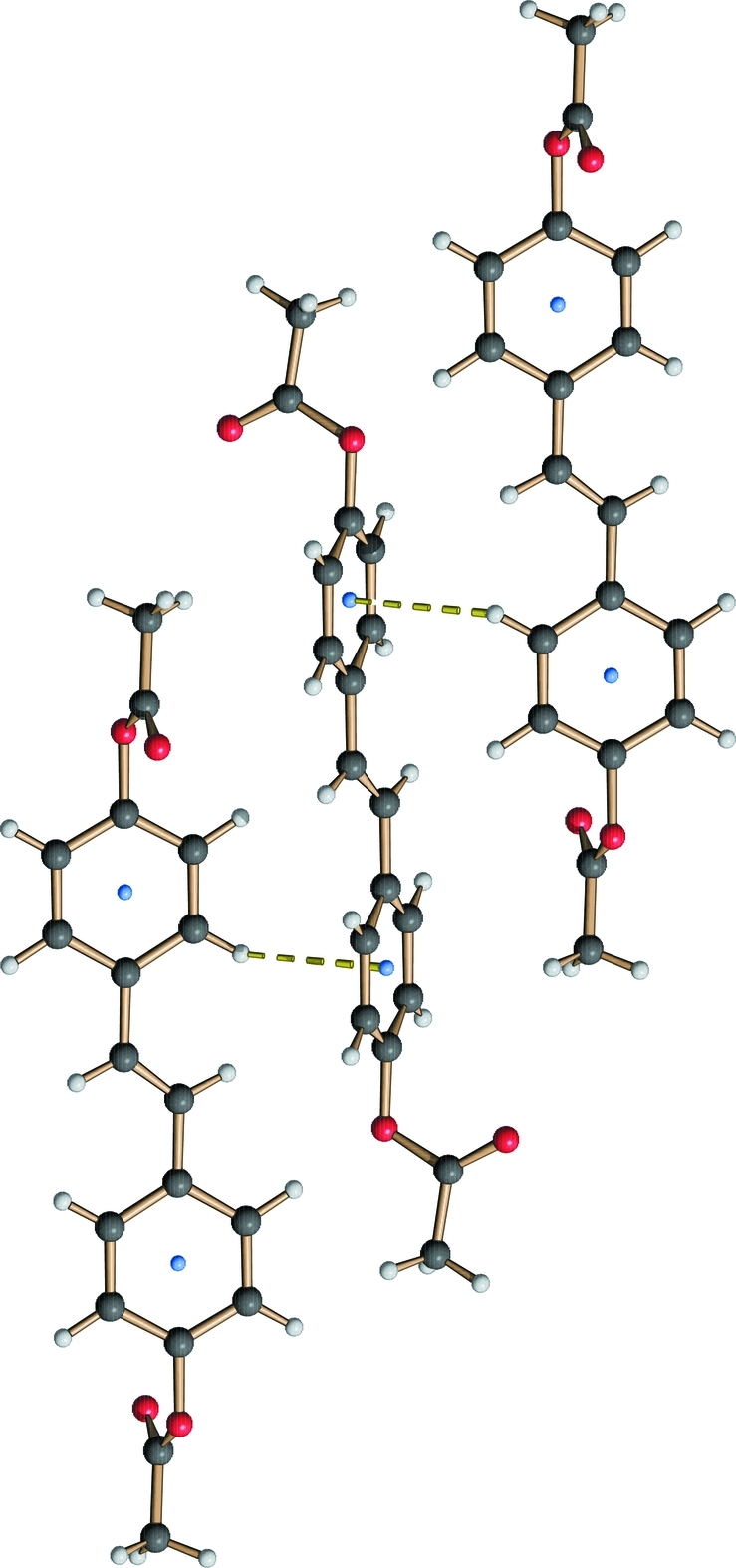
Intramolecular C—H···π interactions.

## supplementary crystallographic information

### Comment

Resveratrol-related stilbenes exhibit promising anticancer activity (Aggarwal *et al.*, 2004; Wolter *et al.*, 2002; Fremont *et al.*, 2000; Jang *et al.*, 1997). Based on our own research in the field of bioactive stilbenes (Wieder *et al.*, 2001) we decided to reinvestigate the possibility of using a cross-metathesis strategy for the synthesis of compounds of type 1 (Velder *et al.*, 2006) which turned out to be a highly efficient route towards symmetrically as well as unsymmetrically substituted E-stilbenes. Alternative strategies for the synthesis of stilbenes are based on Wittig-type olefinations or Heck couplings (Kim *et al.* (2002), Lion *et al.* (2005), Botella *et al.* (2004), Reetz *et al.* (1998)). One of the compounds prepared is the title compound *trans*-1,2-bis-(4-acetoxyphenyl)ethene. Within each molecule the two planes defined by the arene moieties are co-planar but slightly stepped (by 0.324 (2) Å) due to the fact that the plane defined by the central double bond is twisted by a torsion angle of -13.8 (2)° (C1a—C1—C2—C7) and 165.7 (15)° (C1a—C1—C2—C3), respectively (figure 1). The molecules form layers which are intermolecularly linked through a C—H···π interaction of type III (Malone *et al.* 1997). This interaction occurs between the H atom of one phenyl group and the π-system of the other phenyl moiety (figure 2). With a H···π distance of only 2.77 Å these interactions are rather strong.

### Experimental

In a glove-box (Labmaster 130, mBraun), the catalyst (Grubbs-II, 2 mol %) was weighted into a 25 ml Schlenk tube, which was sealed with a rubber septum. This was then taken out of the box, connected to an Ar-vacuum double manifold and equipped with a reflux condenser under argon. A solution of 3-acetoxy-styrene (1.0 g, 6.17 mmol) in CH_2_Cl_2_ (20 ml) was added *via* syringe and the resulting solution was refluxed for 1.5 h under argon. After allowing the reaction mixture to cool to room temperature, the solvent was evaporated *in vacuo* and the crude product was purified by recrystallization from EtOAc/cyclohexane 5:1 to give 0.8 g (88%) of the homo-metathesis product 1. mp. 214 °C (Johnson *et al.* (1952) 215–218°C). ^1^H NMR (300 MHz, CDCl_3_): δ = 2.29 (s, 3H, CH_3_), 7.04 (s, 1H, CH=), 7.08 (d, 2H, J = 8.7 Hz, H-3, H-5), 7.49 (d, 2H, J = 8.7 Hz, H-2, H-6); ^13^C NMR (300 MHz, CDCl_3_): δ = 21.2 (CH_3_), 121.8 (C-3, C-5), 127.4 (C-2, C-6), 127.9 (C-7), 135.0 (C-1), 150.1 (C-4), 169.5 (C=O); HRMS, calcd for C_18_H_16_O_4_ (*M*^+^) 296.1048, found 296.105.

### Refinement

Hydrogen atoms were located in difference syntheses, and are refined at idealized positions (C—H = 0.98Å for methyl H atoms and 0.95Å for all other H Atoms) using a riding model, the U values of the H atoms are constrained relative to *U*_eq_ of the parent carbon atom (1.2 *x U*_eq_(C) for C—H and 1.5 *x U*_eq_(C) for methyl H).

### Crystal data

**Table:**

C_18_H_16_O_4_	*F*(000) = 312
*M**_r_* = 296.31	*D*_x_ = 1.343 Mg m^−^^3^
Monoclinic, *P*2_1_/*c*	Melting point: 214 K
Hall symbol: -P 2ybc	Mo *K*α radiation, λ = 0.71073 Å
*a* = 9.7430 (4) Å	Cell parameters from 3533 reflections
*b* = 7.2839 (4) Å	θ = 2.3–27.0°
*c* = 11.2723 (6) Å	µ = 0.10 mm^−^^1^
β = 113.649 (3)°	*T* = 100 K
*V* = 732.78 (7) Å^3^	Needle, colourless
*Z* = 2	0.52 × 0.36 × 0.34 mm

### Data collection

**Table:**

Nonius KappaCCD diffractometer	1119 reflections with *I* > 2σ(*I*)
Radiation source: fine-focus sealed tube	*R*_int_ = 0.038
graphite	θ_max_ = 27.0°, θ_min_ = 2.3°
φ and ω scans	*h* = −12→12
3533 measured reflections	*k* = −8→9
1595 independent reflections	*l* = −14→14

### Refinement

**Table:**

Refinement on *F*^2^	Primary atom site location: structure-invariant direct methods
Least-squares matrix: full	Secondary atom site location: difference Fourier map
*R*[*F*^2^ > 2σ(*F*^2^)] = 0.042	Hydrogen site location: inferred from neighbouring sites
*wR*(*F*^2^) = 0.106	H-atom parameters constrained
*S* = 1.02	*w* = 1/[σ^2^(*F*_o_^2^) + (0.0486*P*)^2^ + 0.0561*P*] where *P* = (*F*_o_^2^ + 2*F*_c_^2^)/3
1595 reflections	(Δ/σ)_max_ = 0.002
101 parameters	Δρ_max_ = 0.20 e Å^−^^3^
0 restraints	Δρ_min_ = −0.21 e Å^−^^3^

### Special details

**Table:**

Geometry. All e.s.d.'s (except the e.s.d. in the dihedral angle between two l.s. planes) are estimated using the full covariance matrix. The cell e.s.d.'s are taken into account individually in the estimation of e.s.d.'s in distances, angles and torsion angles; correlations between e.s.d.'s in cell parameters are only used when they are defined by crystal symmetry. An approximate (isotropic) treatment of cell e.s.d.'s is used for estimating e.s.d.'s involving l.s. planes.
Refinement. Refinement of *F*^2^ against ALL reflections. The weighted *R*-factor *wR* and goodness of fit *S* are based on *F*^2^, conventional *R*-factors *R* are based on *F*, with *F* set to zero for negative *F*^2^. The threshold expression of *F*^2^ > σ(*F*^2^) is used only for calculating *R*-factors(gt) *etc*. and is not relevant to the choice of reflections for refinement. *R*-factors based on *F*^2^ are statistically about twice as large as those based on *F*, and *R*- factors based on ALL data will be even larger. The coordinates of the hydrogen atoms are constrained, and the U values of the H atoms are constrained relative to the *U*_eq_ of the atom the hydrogen binds to (1.2 for CH and CH_2_, 1.5 for CH_3_).

### Fractional atomic coordinates and isotropic or equivalent isotropic displacement parameters (Å^2^)

**Table:**

	*x*	*y*	*z*	*U*_iso_*/*U*_eq_	
O1	0.26496 (12)	0.47976 (14)	0.58904 (9)	0.0210 (3)	
O2	0.37476 (12)	0.75603 (15)	0.60504 (10)	0.0249 (3)	
C1	0.06877 (16)	0.48067 (19)	0.04361 (14)	0.0165 (3)	
H1	0.1412	0.4466	0.0111	0.020*	
C2	0.11855 (16)	0.48580 (19)	0.18473 (14)	0.0151 (3)	
C3	0.25433 (16)	0.4033 (2)	0.26319 (14)	0.0171 (3)	
H3	0.3134	0.3468	0.2238	0.020*	
C4	0.30488 (16)	0.4019 (2)	0.39715 (14)	0.0176 (3)	
H4	0.3971	0.3446	0.4492	0.021*	
C5	0.21842 (17)	0.4853 (2)	0.45310 (13)	0.0165 (4)	
C6	0.08410 (17)	0.5691 (2)	0.37923 (14)	0.0189 (4)	
H6	0.0261	0.6259	0.4195	0.023*	
C7	0.03502 (17)	0.5694 (2)	0.24605 (14)	0.0179 (4)	
H7	−0.0572	0.6274	0.1950	0.021*	
C8	0.34023 (17)	0.6307 (2)	0.65644 (15)	0.0187 (4)	
C9	0.37163 (18)	0.6128 (2)	0.79670 (14)	0.0245 (4)	
H9A	0.4210	0.7245	0.8424	0.037*	
H9B	0.4373	0.5070	0.8330	0.037*	
H9C	0.2773	0.5951	0.8069	0.037*	

### Atomic displacement parameters (Å^2^)

**Table:**

	*U*^11^	*U*^22^	*U*^33^	*U*^12^	*U*^13^	*U*^23^
O1	0.0304 (7)	0.0177 (6)	0.0123 (6)	−0.0031 (5)	0.0058 (5)	−0.0012 (4)
O2	0.0264 (7)	0.0227 (6)	0.0258 (6)	−0.0058 (5)	0.0106 (5)	−0.0025 (5)
C1	0.0191 (8)	0.0139 (8)	0.0174 (8)	−0.0008 (6)	0.0083 (6)	−0.0011 (6)
C2	0.0168 (8)	0.0117 (7)	0.0154 (8)	−0.0031 (6)	0.0051 (6)	0.0008 (6)
C3	0.0184 (8)	0.0156 (8)	0.0175 (8)	−0.0012 (6)	0.0076 (7)	−0.0019 (6)
C4	0.0156 (8)	0.0154 (8)	0.0181 (8)	−0.0004 (6)	0.0027 (6)	0.0013 (6)
C5	0.0239 (9)	0.0132 (8)	0.0106 (8)	−0.0049 (6)	0.0049 (7)	−0.0005 (6)
C6	0.0245 (9)	0.0145 (8)	0.0192 (8)	0.0008 (6)	0.0102 (7)	−0.0023 (6)
C7	0.0198 (9)	0.0152 (8)	0.0169 (8)	0.0018 (6)	0.0055 (7)	0.0010 (6)
C8	0.0146 (8)	0.0187 (9)	0.0212 (8)	0.0035 (6)	0.0056 (7)	−0.0039 (7)
C9	0.0275 (9)	0.0245 (9)	0.0174 (9)	0.0029 (7)	0.0046 (7)	−0.0037 (7)

### Geometric parameters (Å, °)

**Table:**

O1—C8	1.3700 (18)	C4—C5	1.380 (2)
O1—C5	1.4135 (16)	C4—H4	0.9500
O2—C8	1.1994 (17)	C5—C6	1.380 (2)
C1—C1^i^	1.336 (3)	C6—C7	1.381 (2)
C1—C2	1.466 (2)	C6—H6	0.9500
C1—H1	0.9500	C7—H7	0.9500
C2—C3	1.398 (2)	C8—C9	1.491 (2)
C2—C7	1.401 (2)	C9—H9A	0.9800
C3—C4	1.388 (2)	C9—H9B	0.9800
C3—H3	0.9500	C9—H9C	0.9800
			
C8—O1—C5	116.48 (11)	C5—C6—C7	119.19 (14)
C1^i^—C1—C2	126.23 (17)	C5—C6—H6	120.4
C1^i^—C1—H1	116.9	C7—C6—H6	120.4
C2—C1—H1	116.9	C6—C7—C2	121.30 (14)
C3—C2—C7	117.66 (14)	C6—C7—H7	119.3
C3—C2—C1	119.52 (13)	C2—C7—H7	119.3
C7—C2—C1	122.81 (13)	O2—C8—O1	122.30 (14)
C4—C3—C2	121.62 (14)	O2—C8—C9	126.94 (14)
C4—C3—H3	119.2	O1—C8—C9	110.76 (13)
C2—C3—H3	119.2	C8—C9—H9A	109.5
C5—C4—C3	118.61 (14)	C8—C9—H9B	109.5
C5—C4—H4	120.7	H9A—C9—H9B	109.5
C3—C4—H4	120.7	C8—C9—H9C	109.5
C4—C5—C6	121.62 (13)	H9A—C9—H9C	109.5
C4—C5—O1	119.59 (13)	H9B—C9—H9C	109.5
C6—C5—O1	118.75 (13)		
			
C1^i^—C1—C2—C3	165.70 (18)	C8—O1—C5—C6	−85.05 (16)
C1^i^—C1—C2—C7	−13.8 (3)	C4—C5—C6—C7	0.0 (2)
C7—C2—C3—C4	0.6 (2)	O1—C5—C6—C7	−177.66 (13)
C1—C2—C3—C4	−178.87 (13)	C5—C6—C7—C2	0.3 (2)
C2—C3—C4—C5	−0.4 (2)	C3—C2—C7—C6	−0.6 (2)
C3—C4—C5—C6	0.1 (2)	C1—C2—C7—C6	178.93 (13)
C3—C4—C5—O1	177.71 (12)	C5—O1—C8—O2	−5.1 (2)
C8—O1—C5—C4	97.22 (15)	C5—O1—C8—C9	175.46 (12)

Symmetry codes: (i) −*x*, −*y*+1, −*z*.

### Hydrogen-bond geometry (Å, °)

**Table:**

*D*—H···*A*	*D*—H	H···*A*	*D*···*A*	*D*—H···*A*
C7—H7···Cg1^ii^	0.95	2.81	3.539 (2)	135

Symmetry codes: (ii) −*x*, *y*+1/2, −*z*+1/2.
